# The inhibitory potential of green manure return on the germination and seedling growth of *Eleusine indica L.*

**DOI:** 10.3389/fpls.2024.1287379

**Published:** 2024-02-07

**Authors:** Ying Zhang, Silin Liu, Xiao Du, Zhongwen Chen, Zhiyu Ma, Yinghui Mu

**Affiliations:** ^1^ College of Agriculture, South China Agricultural University, Guangzhou, China; ^2^ College of Natural Resources and Environment, South China Agricultural University, Guangzhou, China; ^3^ School of Electrical and Mechanical Engineering, Zhongkai University of Agriculture and Engineering, Guangzhou, China; ^4^ College of Engineering, South China Agricultural University, Guangzhou, China; ^5^ Scientific Observing and Experimental Station of Crop Cultivation in South China, College of Agronomy/Ministry of Agriculture and Rural Affairs, Guangzhou, China

**Keywords:** green manure, decomposition, weed controlling, seed viability, photosynthetic parameters, antioxidant enzyme activity

## Abstract

*Trifolium repens* L. (white clover) and *Lolium perenne* L. (ryegrass) are green manures widely used in conservation tillage systems worldwide. *Eleusine indica* L. (goosegrass) is a globally recognized noxious weed. Herein, we investigated the effects of aqueous extracts, decomposed liquids, and different straw-to-soil ratios on the germination and growth of goosegrass. The results showed that high concentrations (≥ 30%) of aqueous extracts or decomposed liquids of both green manures significantly inhibited germination-related parameters of goosegrass. The strongest inhibitory effect was observed for the 7-day decomposition treatment, and white clover’s inhibitory effect was greater than ryegrass’s. A pot experiment showed that non-photochemical quenching, catalase, and peroxidase activity levels of goosegrass leaves were significantly increased. At the same time, the net photosynthetic rate significantly decreased. Seedling growth was inhibited when the straw-to-soil ratio was greater than 3:100. The ryegrass treatments inhibited goosegrass seedlings more than the white clover treatments. This study demonstrated the inhibitory potential of white clover and ryegrass straw return on seed germination and seedling growth of goosegrass. The study has also helped to identify weed-resistant substances in these green manures so that their weed-control properties can be used more effectively and herbicide usage can be reduced.

## 1 Introduction

Herbicides are essential for agricultural output because weeds pose a serious threat to many agricultural systems. However, decades of herbicide usage have caused a rise in the number of herbicide-resistant weed populations and their toxicity to the environment, including crops, air, water, and soil (James et al., 2018; Tudi et al., 2021; Tataridas et al., 2022). High glyphosate (i.e., a common herbicide) levels in rivers and seas in China have been reported (Devi et al., 2022). There is a real and pressing demand to reduce the usage of herbicides. Planting green manures, also known as cover crops, effectively controls weed growth in farmlands (Mirsky et al., 2011). Green manures can suppress weed growth because they occupy the same ecological niche as weeds and compete with them for resources such as light, nutrients, water, etc. (Valentin et al., 2017). Additionally, they can release allelochemicals to inhibit or delay the germination of some weeds via allelopathy (Scepanovic et al., 2021; Tang et al., 2022).

Allelopathy has been defined as the chemical mediation of plant interactions. Allelochemicals, which are generally plant secondary metabolites, are released into the environment by foliar leaching, volatilization, residue decomposition, and root exudation and can inhibit the germination, growth, and survival of neighboring plants (Rice, 1985; Warren et al., 2017; Li et al., 2021). In a concentration-dependent manner, allelochemicals may significantly reduce germination and seedling growth in neighboring plants (Wang et al., 2018; Zhu et al., 2020; Wang et al., 2022). Furthermore, at the early stages of residue decomposition, the levels of inhibitory allelochemicals can be highly phytotoxic; however, as residue decomposition time increases, the levels eventually decrease (Puig et al., 2018). Many studies have attempted to design and develop herbicides based on major allelochemicals, such as phenolic compounds, terpenoids, and alkaloids, because of the inhibitory effects of these compounds (Macías et al., 2010; de Albuquerque et al., 2011; Jabran et al., 2015; Macías et al., 2019).

Conservation tillage is widely used in agricultural production worldwide because it improves soil health, stabilizes and increases crop yields, reduces greenhouse gas emissions, and so on (Derpsch et al., 2014; Dat and Lyubov, 2019; Deng et al., 2022). Conservation tillage can also control weeds by retaining crop residues to restrict the growth and propagation space for the weeds, increasing the populations of weed seed predators, combining crop rotation, etc. (Derpsch et al., 2014; Quinn et al., 2016; Deng et al., 2022; Yu et al., 2022). The numbers of Chinese research and applications concerning conservation tillage in northern areas of China are more prevalent than those in southern areas (Ao et al., 2021). It is theoretically feasible to use green manures and conservation tillage to suppress weeds in southern China, which has abundant water and heat resources and a serious weed threat (China Geological Survey, 2015).

White clover (WC) is a fast-growing perennial legume herb with intense coverage. Ryegrass (RG) is a multi-tillering herb in the Gramineae family that grows quickly. These plants are multipurpose green manures that are used widely worldwide (Bergtold et al., 2019). Goosegrass is an annual gramineous weed that occurs worldwide and harms crop yields (Zhang et al., 2021). It has a very high population in southern China because of the high temperatures and rainfall in the region. In our previous study, these green manures showed inhibitory effects on weed biomass in “green manure–maize–peanut” rotation systems, although the impacts of allelopathic effects have not been studied (Liu et al., 2022). The present study focuses on the effects of WC and RG straw on the germination and seedling growth of goosegrass, employing aqueous extracts (AE), decomposed liquids (DL), and straw return experiments to provide a scientific basis for using green manures to reduce herbicide usage.

## 2 Materials and methods

### 2.1 Plant material

Wild goosegrass seeds were collected at the Zengcheng Experimental Base of South China Agricultural University (23°24′29.85″N, 113°64′39.06″E). Green manures for the trials were sown in November 2019 and collected at the base in March 2020. The source of seeds was Junji Garden Seedling Farm. The collected straw was cleaned with clean water, allowed to dry naturally, crushed, and stored at indoor temperatures for later use.

### 2.2 Experimental and treatment design

**Figure 1:**
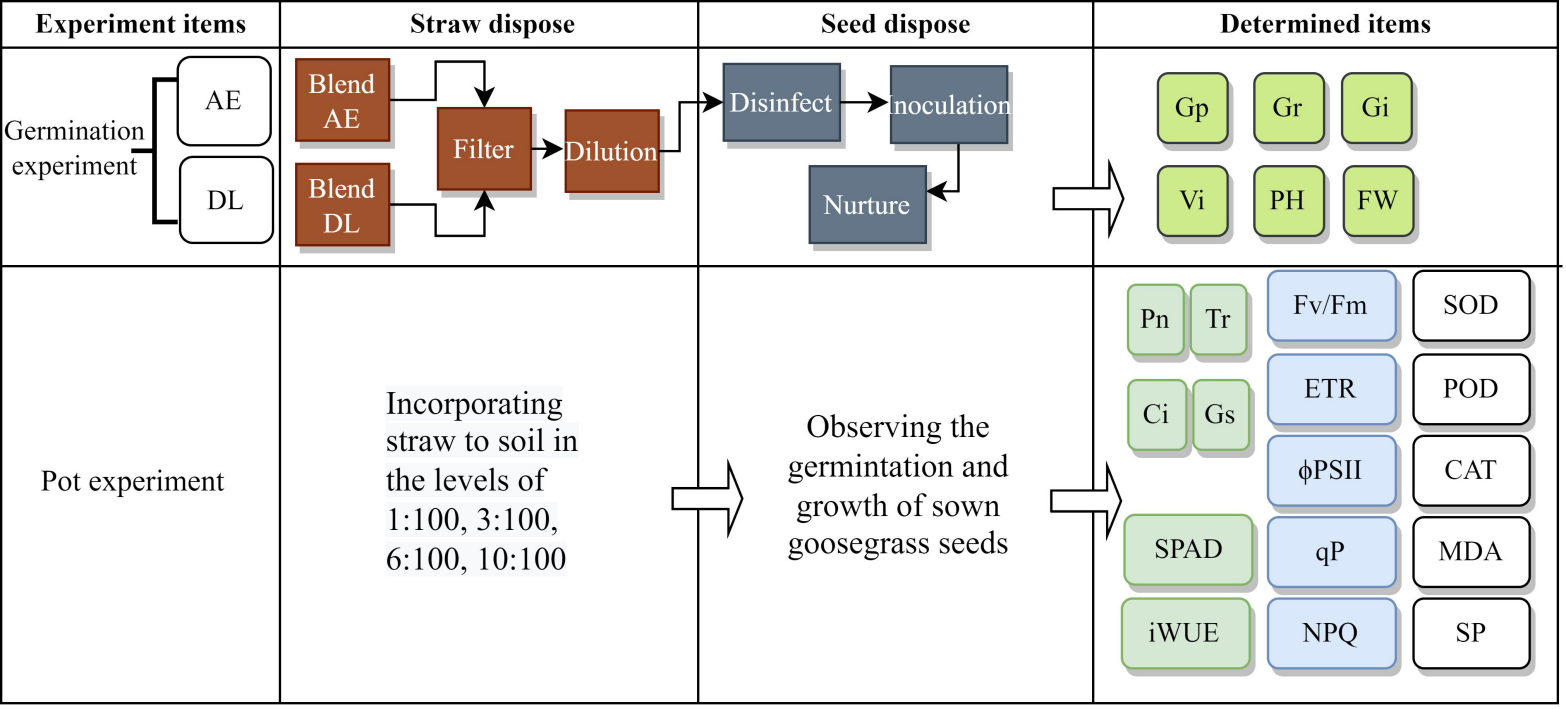
The flowchart of the experimental process (WC, white clover; RG, ryegrass; AE, aqueous extract; DL: decomposed liquid).

The abbreviations appearing throughout **Figure 1** are in the **Supplementary Material** (**Table S1**).

We used dry WC and RG powder as the materials. In the AE experiment, 20 g of material were weighed to be mixed with 500 mm of sterilized water in a bottle and soaked in a shaker for 48 h at 200 rpm. This extract was filtered sequentially with filter paper and centrifuged at 12,000 rpm for 10 minutes. A 0.22 μm microporous membrane filtered the supernatant to remove bacteria. A 100% concentration of AE was prepared. The extracts were diluted with sterilized ultra-pure water into 2, 5, 30, and 80% of the treatment solution. Sterile water treatment was used as a control. Each treatment was set up in five replicates. We used 2.2% sodium hypochlorite to disinfect the goosegrass seeds for ten minutes, adopted the double-layer filter paper method, inoculated 30 goosegrass seeds into Petri dishes, and treated them with the treatment solution. The inoculated Petri dishes were placed in an artificial climate incubator at a temperature of 26 ± 2°C, a relative humidity of 75%, and a light-dark cycle of 12 h/12 h. The water was replenished and the germination number was recorded every day. The emergence of the radicle indicated the germination of goosegrass. The recording was stopped when the germination rate remained unchanged for three days.

In the decomposition experiment, we mixed material, fresh paddy soil, and ultra-pure water according to a weight ratio of 1:1:30 and put them into a plastic bottle with an inner lid. A soil solution without powder was used as the control. The mixtures were placed in a constant temperature shaker at 28°C for 200 rpm for 2, 3, 7, and 15 days. The extracts were sequentially filtered with filter paper, centrifuged (with the program set at 12,000 rpm for 10 min), and filtered with a 0.22 μm microporous membrane. A 100% concentration of DL was prepared. The next steps were similar to the AE experiment.

In the pot experiment, we mixed WC or RG powder with the substance (consisting of peat soil, coconut bran, vermiculite, and perlite) at a mass ratio of 1:100, 3:100, 6:100, and 10:100 (Liu et al., 2022). The substance without material was the control. The mixtures were put in the flowerpot, and 30 goosegrass seeds were sown. Each treatment was set up in five replicates.

### 2.3 Test items

Germinated seeds were counted daily. Goosegrass seeds were scored as germinated when their radicle length reached 1 mm. The plant height (PH) was measured from 10 seedlings with uniform growth selected from each treatment on the seventh day after inoculation. The fresh weight (FW) was measured for 20 seedlings randomly selected from 150 seeds from each treatment for three replicates (a particular proportion reduced the number of samples, and the final result was multiplied by the corresponding multiple after weighing when the sample was insufficient).


$$Gp=\frac{number\;of\;germinated\;seeds\;at\;2nd\;day}{number\;of\;tested\;seeds}$$

$$Gr=\frac{number\;of\;normal\;germinated\;seeds\;at\;7th\;day}{number\;of\;tested\;seeds}$$

$$Gi=\sum \frac{Gt}{Dt}$$

Vi=PH×G;i

where Gr is the germination rate, Gp is the germination potential, Gi is the germination index, Gt is the germination number on different days, Dt is the statistical number of days, and Vi is the vital index.

The allelopathy response index (RI) was evaluated to determine the allelopathic effects of the AE and DL on goosegrass germination and seedling growth. The RI was defined as 1−C/T if T ≥ C or as T/C−1 if T< C, where C and T are the control and treatment values, respectively (Bruce and Richardson, 1988). RI > 0 indicates allelopathic stimulation, and RI< 0 indicates allelopathic inhibition. The absolute value was consistent with the degree of allelopathy. RI accumulate value = RI_Gr_ + RI_Gp_ + RI_Gi_ + RI_Vi_ + RI_PH_ + RI_FW_. The RI accumulated value reached −6, indicating that germination was fully inhibited.

In the pot experiment, the last fully expanded goosegrass seedling leaf was selected for analysis from five replicates of each treatment. Superoxide dismutase (SOD) activity was measured by the nitro tetrazolium blue chloride reduction method, peroxidase (POD) activity by a colorimetric method, and catalase (CAT) activity by the guaiacol method (Zhang et al., 2017). The soluble protein (SP) content was measured by Coomassie brilliant blue staining (Jamshidi et al., 2021). The malondialdehyde (MDA) content was measured by the thiobarbituric acid method (Xiao et al., 2021).

The LI-6,800 photosynthesis measurement system (LI-COR, United States) was used to measure the following photosynthesis and fluorescence-related indexes: the net photosynthetic rate (Pn), the transpiration rate (Tr), stomatal conductance (Gs), the intercellular carbon dioxide concentration (Ci), the instant water use efficiency (iWUE), the maximal quantum yield of photosystem II (Fv/Fm), the quantum yield of photosystem II (ΦPSII), the electron transport rate (ETR), photochemical quenching (qP), and non-photochemical quenching (NPQ). The SPAD values (a leaf color value used to describe the relative chlorophyll content) were measured by a SPAD-502 plus (KONICA MINOLTA, Japan) instrument, which calculated the average of the leaf’s upper, middle, and lower parts.

### 2.4 Statistical analyses

The experimental data were analyzed using a one-way ANOVA. A two-way ANOVA was used to assess two main factors and their interactions: species and concentration in the AE experiment, decomposition times and concentration in the DL experiment, and species and ratios in the pot experiment. Significant differences were further compared using the *post hoc* Fisher LSD test. The significance analysis of the data was assessed in SPSS 24, plotted in GraphPad Prism 7, and expressed as the mean ± standard deviation. Correlation analysis, principal component analysis, and their visualizations were performed in RStudio 2023.03.0 (Posit Software, Boston, Massachusetts). The flowchart was plotted in draw.io v. 20.3.0 (JGraph Ltd., Northampton, England).

## 3 Results

### 3.1 Effects of aqueous extracts of green manures on goosegrass seed germination and seedling growth

**Figure 2:**
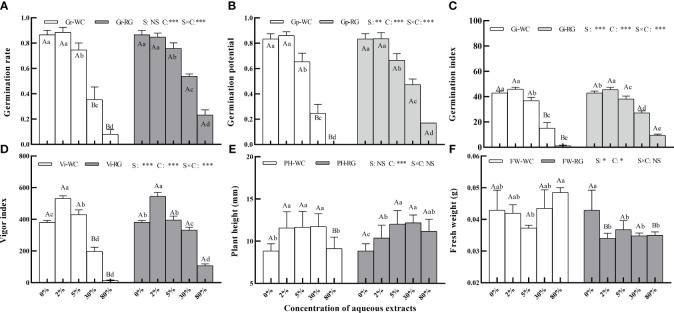
Effects of the AE concentration of green manure species on goosegrass seed germination (**A–D**, n = 5), plant height (**E**, n = 10), and fresh weight (**F**, n = 3). Data are presented as mean ± SD. Different capital letters indicate significant differences between green manure species by the same concentration (P<0.05). Lowercase letters indicate significant differences among extract concentrations by the same green manures (P ≤ 0.05). S: green manures species; C: concentration; *: P ≤ 0.05; **: P ≤ 0.01; ***: P ≤ 0.001; NS: no significant difference.

Primary treatment factors of green manure species (S), concentration (C), and their interactions (S × C) significantly affected goosegrass seed Gp, Gi, and Vi (**Figure 2**, P ≤ 0.01). The factors C and S × C had the same effect on Gr except for S. Low concentrations of AE from the two green manures stimulated Gr, Gp, Gi, and Vi, while higher concentrations caused inhibition. It had a significantly inhibitory effect on the Gr, Gp, and Gi of both when the AE concentration reached 5%. At 30% concentration, WC had a stronger inhibitory effect than RG (**Figures 2A–D**, P ≤ 0.05). Only factor C significantly affected the plant height and FW of goosegrass. Both AEs stimulated seedling height (**Figures 2E, F**).

### 3.2 Effects of decomposed liquids of green manures on goosegrass seed germination and seedling growth

**Figure 3:**
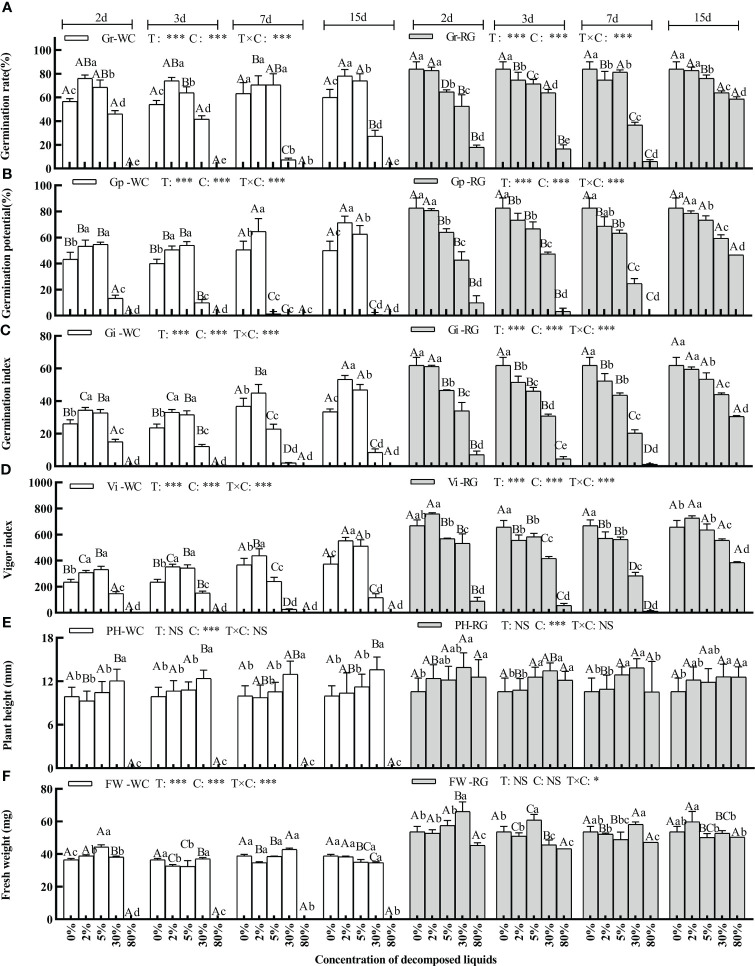
Effects of the DL concentration of green manure species on goosegrass seed germination (**A–D**, n = 5), plant height (**E**, n = 10), and fresh weight (**F**, n = 3). Data are presented as mean ± SD. Different capital letters indicate significant differences among decomposition days by the same green manure and concentration (P<0.05). Lowercase letters indicate significant differences among extract concentrations by the same green manures and decomposition days (P<0.05). T: decomposition duration; C: concentration; *: P ≤ 0.05; **: P ≤ 0.01; ***: P ≤ 0.001; NS: no significant difference.

The primary factors, decomposition duration (T) and concentration (C), and the interaction (T × C), significantly affected four germination indexes of goosegrass seeds (**Figures 3A–D**, P ≤ 0.001). The same trends as for AE were seen for the DL of the two green manures, i.e., Gr, Gp, Gi, and Vi were stimulated at low concentrations but inhibited at higher concentrations. The four germination indexes of goosegrass seeds under RG-DL were inhibited significantly at a 5% concentration, while the WC treatments had similar effects at a 30% concentration. The goosegrass seeds did not germinate when the WC-DL concentration reached 80% (**Figures 3A–D**, P ≤ 0.05). The inhibitory effects of DL on the four germination indexes increased with increasing duration of decomposition, and the maximum occurred in the treatments containing 7d DL as well as at the 30% concentration. WC and RG had similar effects, but the inhibitory effect of WC was stronger. For WC-DL at a 5% concentration allowed to decompose for 7d, Gp even approached zero (1.33%) (**Figure 3B**). However, Gr under the same conditions was inhibited compared to the control. Only factor C significantly affected the plant height (**Figure 3E**, P< 0.001). Nearly all WC-DL stimulated the plant height except those at an 80% concentration (**Figure 3F**).

### 3.3 Effect of white clover and ryegrass extracts on goosegrass seed germination and seedling growth

**Figure 4:**
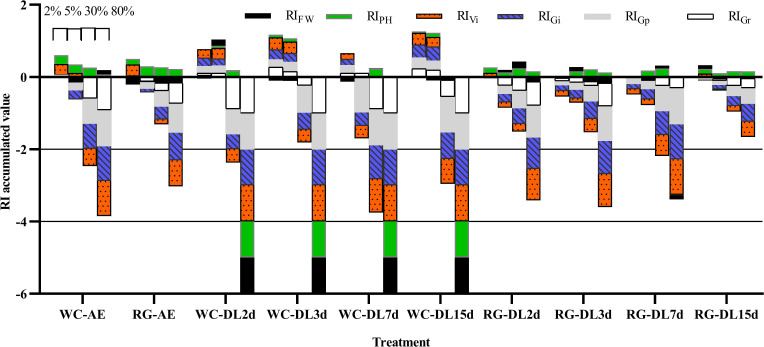
RI accumulated the value of green manure extracts on goosegrass. RI, response index; WC, white clover; RG, ryegrass; AE, aqueous extract; DL, decomposed liquid. The adjacent columns from left to right in the same treatment represent concentrations of 2%, 5%, 30%, and 80% respectively.

Both AE and DL of WG and RG inhibited goosegrass germination at high concentrations (≥ 30%) and stimulated it at low concentrations (2%) (**Figure 4**). Additionally, they had a specific stimulation effect on the plant height. Overall, the absolute values of RI_Gp_ were greater than RI_Gr_, which showed that AE and DL inhibition effects were stronger for Gp than for Gr. The RI accumulated absolute values of WC were greater than those for RG under the same conditions at concentrations ≥ 30%. The inhibition effect of the DL decomposed for 15 days was lower than that for seven days. The RI accumulated absolute value of 30% WC-DL15d was 12.96% lower than that of 30% WC-DL7d, and the values for RG-DL15d averaged at 56.92% lower than those of RG-DL7d.

### 3.4 Effects of white clover and ryegrass straw return to the soil on seedling growth and the SPAD value

**Figure 5:**
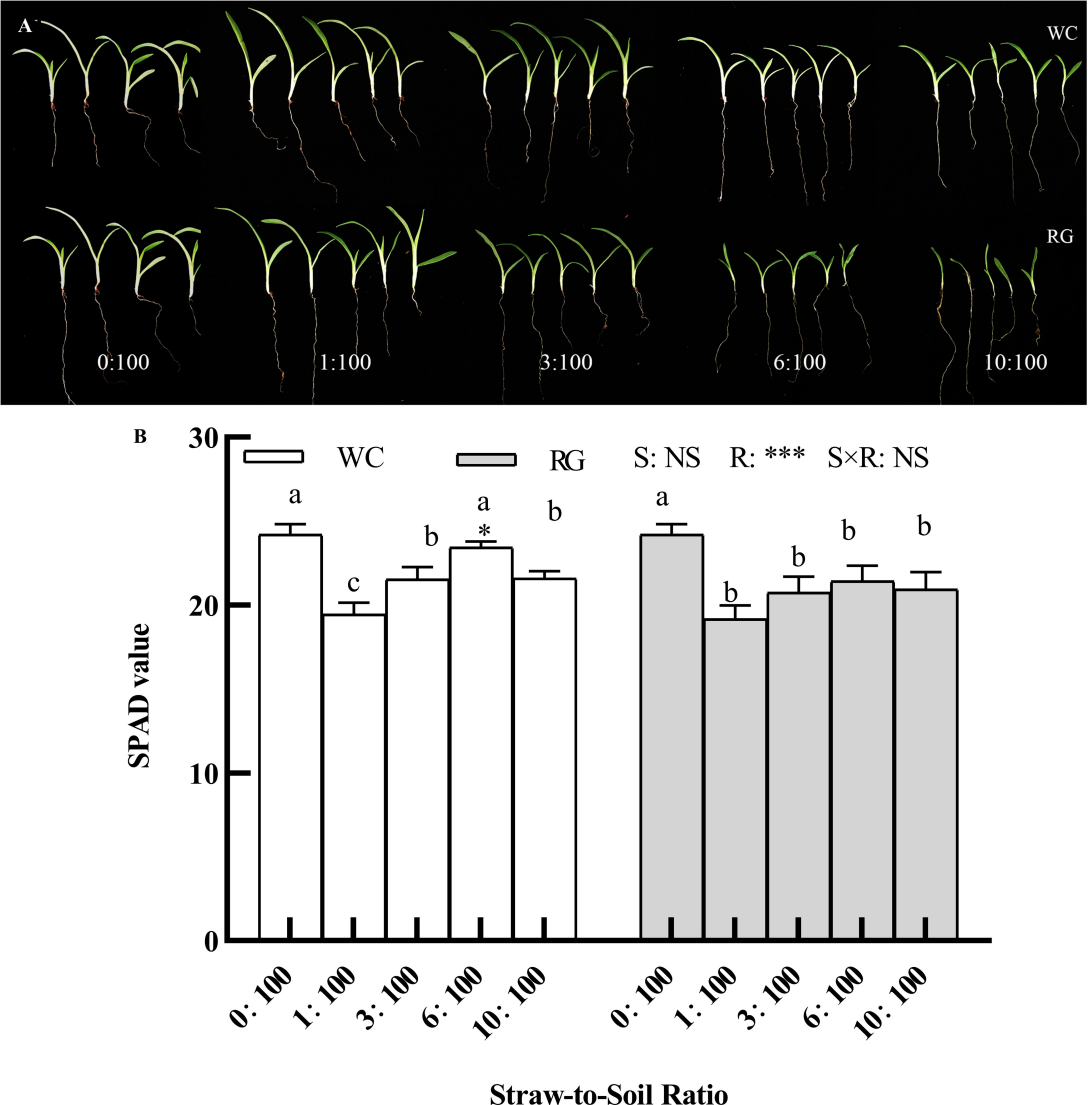
Effect of different ratios on goosegrass seedling growth **(A)** and the SPAD value (**B**, n = 5). Data are presented as mean ± SD. Different lowercase letters indicate significant differences among ratios by the same green manure (P ≤ 0.05). *, **, and *** above the column and the line indicate significant differences between WC and RG by the same ratio at the levels of P ≤ 0.05, P ≤ 0.01, and P ≤ 0.001, respectively. NS: no significant difference. S: green manure species. R: straw-to-soil ratio.

The entire goosegrass seedlings were smaller than those in the 0:100 treatment group, i.e., the control, when the straw-to-soil ratio (SSR) was ≥6:100, and the leaf sizes of the seedlings treated with RG were smaller than those treated with WC (**Figure 5A**). Only the primary factor, the SSR (R), and the interaction (S × R) significantly affected the SPAD value. Nearly all of the treatments significantly inhibited the SPAD of the seedlings except for the WC6:100 treatment, compared with the control (**Figure 5B**).

### 3.5 Effects of white clover and ryegrass straw return to the soil on seedling growth and photosynthesis-related properties of goosegrass

**Figure 6:**
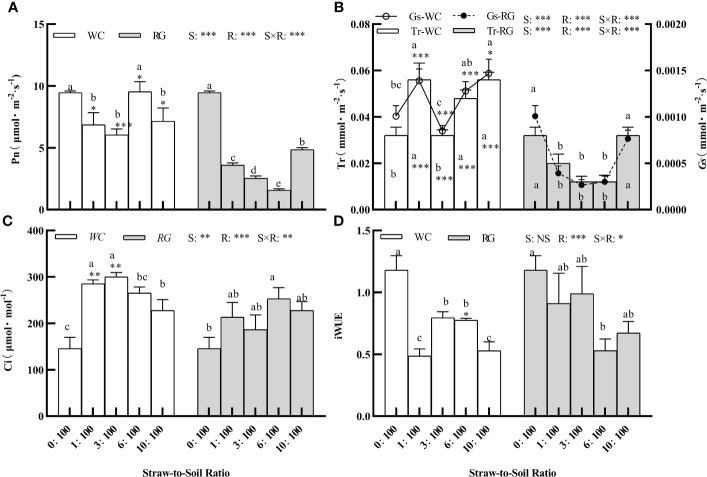
Effect of different ratios on goosegrass photosynthetic parameters (**A–D**, n = 5). Data are presented as mean ± SD. Different lowercase letters indicate significant differences among ratios by the same green manure (P ≤ 0.05). *, **, and *** above the column and the line indicate significant differences between WC and RG by the same ratio P ≤ 0.05, P ≤ 0.01, and P ≤ 0.001, respectively. NS: no significant difference. S: green manure species. R: straw-to-soil ratio.

There was a significant effect among the green manure species (S), R, and the S × R interaction in the goosegrass photosynthetic parameters (i.e., Pn, Gs, Tr, and Ci) (**Figures 6A–D**, P ≤ 0.05). Only R and S × R significantly affected iWUE (**Figure 6B**, P< 0.05). Both the WC and RG treatments suppressed Pn overall compared to the control. The Pn level in the RG treatments was significantly lower than in the WC treatments. It was close to or less than half of the control value (4.73 μmol·m^-2^·s^-1^) when the same ratio was used (**Figure 6A**). WC and RG exhibited similar trends in their effects on Tr and Gs. Compared to the control, the WC treatments significantly stimulated Tr and Gs, while the RG treatments significantly decreased them (**Figure 6B**). Both Ci values were significantly improved under the WC and RG treatments compared to the control. The improvements in the Ci values for the 1:100, 3:100, 6:100, and 10:100 SSR were 95.81%, 106.00%, 82.01%, and 56.20%, respectively, compared to the control (**Figure 6C**). C and RG both significantly inhibited iWUE compared with the control group. With the increase in the ratio, the decreases in WC were 58.79%, 32.59%, 34.39%, and 55.27%, whereas those in RG were 22.86%, 16.17%, 55.13%, and 43.03%, respectively, compared to the control (**Figure 6D**).

### 3.6 Effects of white clover and ryegrass straw return to the soil on fluorescence parameters of goosegrass

**Figure 7:**
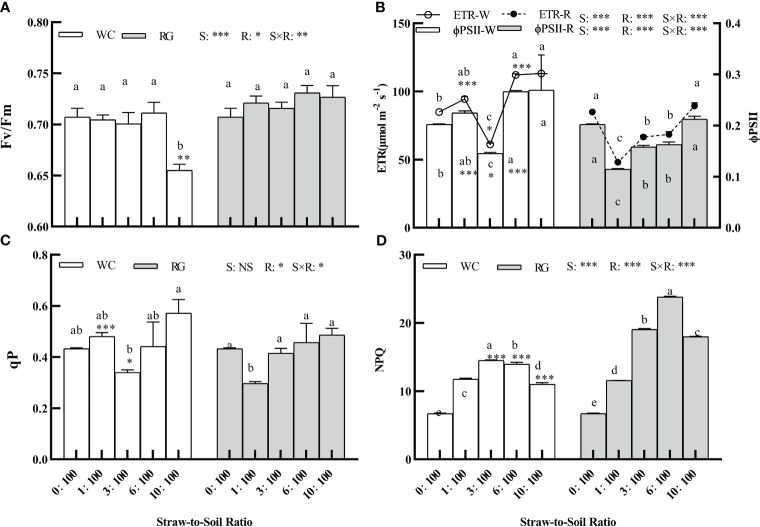
Effect of different ratios on the fluorescence parameters of goosegrass seedlings (**A–D**, n = 5). Data are presented as mean ± SD. Different lowercase letters indicate significant differences between ratios by the same green manure (P ≤ 0.05). *, **, and *** above the column and the line indicate significant differences between WC and RG by the same ratio at P ≤ 0.05, P ≤ 0.01, and P ≤ 0.001, respectively. NS: no significant difference. S: green manure species. R: straw-to-soil ratio.

The factors S, R, and S × R had a significant effect on the goosegrass fluorescence parameters (Fv/Fm, ETR, ΦPSII, and NPQ, P ≤ 0.05; **Figures 7A, B, D**). Only R and S × R significantly affected qP (P ≤ 0.05, **Figure 7C**). Only WC10:100 treatment significantly inhibited Fv/Fm, with a decrease of 7.38% (**Figure 7A**). The WC and RG treatments showed similar trends in their effects on ETR and ΦPSII. Overall, the WC treatment significantly increased ETR and ΦPSII, while RG significantly suppressed them. The WC treatment showed significantly higher ETR and ΦPSII values than RG at the 1:100, 3:100, and 6:100 ratios (**Figure 7B**). Both the WC and RG significantly increased the NPQ compared to the control. The stimulation ranges initially increased and then decreased as the SSR increased. The increases in NPQ for WC were 75.04%, 116.14%, 108.00%, and 64.02%, while those for RG were 72.34%, 183.95%, 254.77%, and 168.01%, respectively, compared to the control. When the ratio was ≥ 3:100, the inhibitory effect of RG was significantly greater than that of WC (**Figure 7D**). Compared to other indicators, qP was less affected by the WC and RG treatments (**Figure 7C**).

### 3.7 Effects of white clover and ryegrass straw returning to the soil on physiology indexes of goosegrass

**Figure 8:**
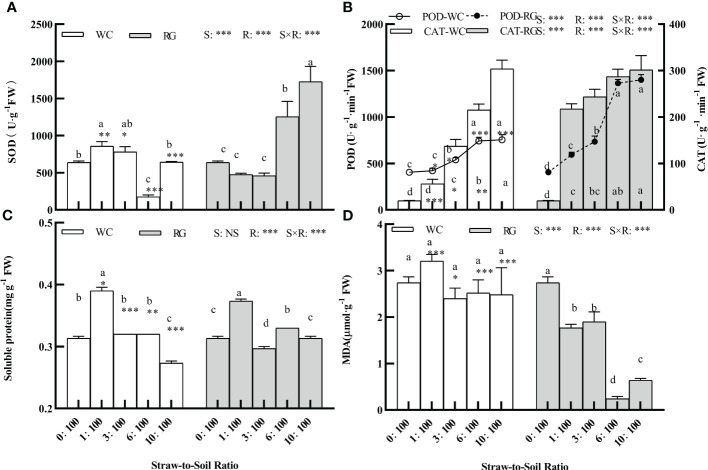
Effects of different ratios on antioxidant enzyme activities **(A, B)**, soluble protein, and MDA content **(C, D)** of goosegrass seedlings. Data are presented as mean ± SD. Different lowercase letters indicate significant differences between ratios by the same green manure (P< 0.05). *, **, and *** above the column and the line indicate significant differences between WC and RG by the same ratio at P< 0.05, P< 0.01, and P< 0.001, respectively. NS: no significant difference. S: green manure species. R: straw-to-soil ratio.

The factors S, R, and S × R interaction significantly affected the SOD, POD, CAT, MDA, and SP values (**Figure 8**, P ≤ 0.01). RG significantly stimulated the physiology indexes of resistance stress in seedlings (i.e., SOD, POD, and CAT). The stimulation ranges by RG were greater than those by WC compared with the control (**Figures 8A, B**, P ≤ 0.05). There was no significant effect on SOD for WC except at the 6:100 ratio. The SP increase for WC and RG only occurred at the 1:100 SSR. No significant effect with WC was seen on the MDA of the goosegrass seedlings, but significant inhibition occurred with RG.

### 3.8 Comprehensive analysis of parameters for the straw return experiment

**Figure 9:**
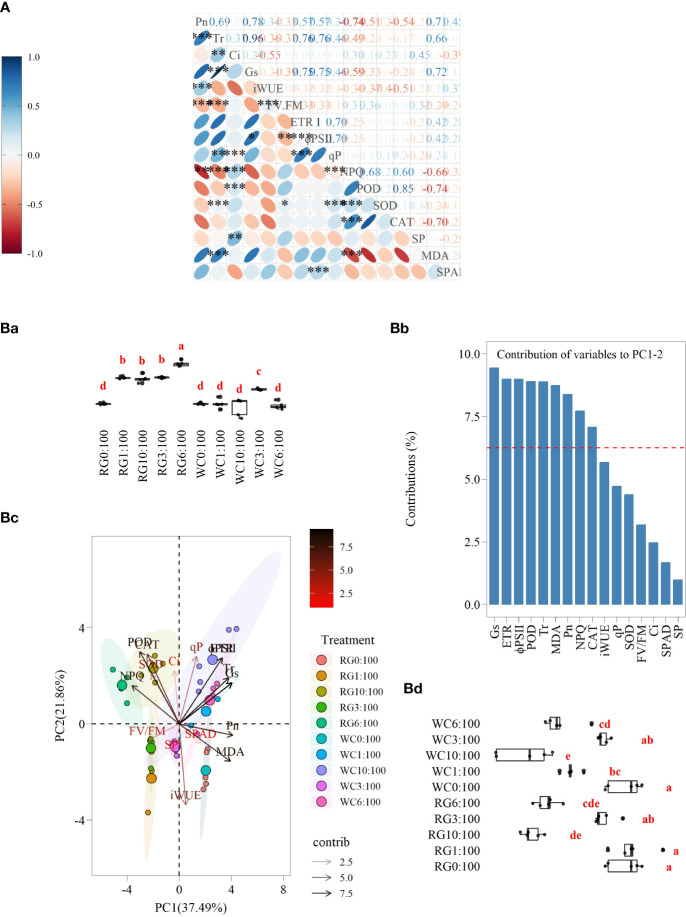
**(A)** Relationship between different parameters of goosegrass seedlings. The red ellipse represents a negative relationship among parameters, and the blue represents a positive relationship among parameters. The stronger the correlation is, the smaller the ellipse is. The numbers represent the correlation coefficients calculated using the Spearman method. ns: no significant difference, * P ≤ 0.05, ** P ≤ 0.01, *** P ≤ 0.001. **(Ba)** The results of multiple comparisons on the eigenvalues of PC1 for different treatments (P ≤ 0.05). **(Bb)** The variance contributions of the 16 parameters in the straw returning experiment to PC1 and PC2. **(Bc)** Principal component analysis (PCA) of different treatments among goosegrass seedlings. **(Bd)** The results of multiple comparisons on the eigenvalues of PC2 for different treatments (P ≤ 0.05).

Pn has the highest number of pairwise correlations (13, r > 0.3) with the other parameters, while parameters Ci, qP, SOD, SP, and SPAD have fewer pairwise correlations (6, 6, 6, 2, and 4, respectively). Specifically, a very high positive correlation exists between Tr and Gs, and ETR and ΦPSII are similar (r > 0.95, P ≤ 0.001). Pn also strongly correlates positively with Tr (r = 0.78, P ≤ 0.001). Pn, Tr, Gs, ETR, ΦPSII, qP, and MDA show positive pairwise correlations between each other. NPQ, POD, and CAT also exhibit similar correlations. Conversely, Pn and MDA negatively correlate with NPQ, POD, and CAT. The negative correlation between Pn and NPQ is highly significant (r = −0.74, P ≤ 0.01). There is a negative relationship between Fv/Fm, Pn, Tr, Gs, ETR, and ΦPSII. It is also significant (P ≤ 0.01) for Pn, Tr, and ΦPSII (**Figure 9A**).

The first two principal components (PC1 and PC2) represent 37.49% and 21.86% of the variation in physiological expression of the goosegrass seedlings, respectively (**Figure 9Bc**). The parameters that show contributions to PC1 and PC2 are greater than expected (i.e., 6.25%), and a total of nine parameters have been ranked in descending order as follows: Gs, ETR, ΦPSII, POD, Tr, MDA, Pn, NPQ, and CAT (**Figure 9Bb**). The angle for the response variables vectors is less than 90 degrees, indicating that they are positively correlated. The angle of negative correlated response variables vectors is greater than 90 degrees. The correlation relationships among the nine main parameters in the PCA analysis are similar to the results of the correlation analysis (**Figure 9Bc**). NPQ, POD, and CAT exhibit positive pairwise correlations. The remaining six parameters also display similar correlations. However, a negative correlation exists between these two groups of parameters. Among the latter group of parameters, the correlation between Pn and MDA is higher than their pairwise correlations with the other four parameters.

The results of the PCA indicate that the nine main parameters are grouped into three categories with respect to their correlations. The first group consists of NPQ, CAT, and POD. The second group includes ETR, ΦPSII, Gs, and Tr. The final group comprises Pn and MDA. When compared to the control, NPQ, CAT, and POD all show similar responses for the WC and RG, that is, a significant enhancement. The size of the increase grows as SSR goes up.

Additionally, NPQ in the RG treatment was significantly higher than in the WC treatment (SSR ≥ 3:100). Overall, Pn exhibited an inhibitory response in the WC and RG; the inhibitory effect was stronger in the RG treatment than in the WC treatment. The WC treatments showed no significant difference in MDA compared to the control, while the RG treatments significantly reduced MDA in the goosegrass seedlings. ETR, ΦPSII, Gs, and Tr showed similar responses in the straw return treatments, but differences were observed between WC and RG. The WC treatment exhibited overall stimulation compared to the control, while RG showed inhibition.

There are significant differences between the RG and WC treatments when it comes to the PC1 variables. Among the WC treatments, only WC3:100 is significantly different from the control (**Figure 9Ba**). For the PC2 variables, significant differences are present between the WC and RG compared to the control overall, and the degree of difference increases with the increase in ratio. The WC and RG treatments only show a significant difference at the 1:100 SSR (**Figure 9Bd**).

## 4 Discussion

High concentrations (≥30%) of white clover and ryegrass extracts have an allelopathic inhibitory effect on the germination of goosegrass seeds. There are many studies using plant extracts to inhibit seed germination to prove their allelopathic inhibitory effect. Wang et al. (2022) found that AE of three herbs inhibited *Lactuca sativa* seeds at high concentrations. Paula et al. (2020) found that the extract and fractions of *Smilax brasiliensis* leaves showed an inhibitory effect on Allium cepa seeds. Gil et al. (2022) found the thermal allelopathic effect of two coniferous plants (*Pinus densiflora* and *P. koraiensis*) on inhibiting *Brassica napus* germination and seedling growth. Sakit et al. (2022) found the phytotoxic effects of *Acacia saligna* stem extract on the germination of economically important crops. The germination experiment showed that goosegrasses for high concentration treatment significantly inhibited gemination indicators, indicating that there was a significantly inhibiting effect on the germination of goosegrass seeds by the extracts (AE and DL) of white clover and ryegrass. The inhibitory effect was greater with the white clover treatment than with the ryegrass treatment.

White clover and ryegrass treatments for high straw-to-soil ratio (≥3:100) had a significant inhibitory effect on the seedling growth of goosegrass. In these conditions, goosegrass seedlings grew more slowly, had lower photosynthesis-related properties, and had higher antioxidant enzyme activities. Studies with regard to the effect of straw return on weeds are usually focused on weed seedbank and weed density (Kumar et al., 2022). We referred to relevant research on plants under stress. Sakit et al. (2022) found that the leachate extract of Acacia saligna decreased photosynthesis, PSII activity, and water use efficiency of tested crop species, with evident effects at higher concentrations. Yan et al. (2022) found that the straw return treatment could reduce the photosynthetic capacity, inhibiting the growth of rice plants. Kumar and Singh (2018) found higher amounts of CAT and SOD in insect-damaged Ludwigia species compared to undamaged plants.

Compared to plants in non-stress conditions, stressed plants had lower photosynthesis and fluorescence parameters and higher antioxidant enzyme activities. However, the pot experiment showed goosegrass seedlings under high straw-to-soil ratios with higher fluorescence parameters (especially NPQ) and lower MDA content compared to the control. Non-photochemical quenching (NPQ) is a protective mechanism that regulates plants’ absorption and dissipation of excess light energy, thereby preventing damage to photosynthetic pigments and chloroplasts (Kreslavski et al., 2007; Ruban, 2016). Accumulation of malondialdehyde (MDA) commonly indicates plants with oxidative damage in stress conditions (Khosrojerdi et al., 2023). We hypothesized that goosegrass seedlings under high ratios with an available fluorescence protective mechanism and without oxidative damage. The treatments for the high ratios of WC and RG only delayed the growth of seedlings. Additionally, the delaying effect was greater with the ryegrass treatment than with the white clover treatment.

We think that it is less possible for the greater delaying effect of the ryegrass treatments under high ratios due to their high fluorescence parameters and low MDA content. Appropriate carbon/nitrogen ratios of green manures can promote straw decomposition (Tang et al., 2021). Previous studies have shown that leguminous green manure return had a greater effect on improving the composition of soil microbial communities than non-leguminous green manure (Zhan et al., 2018), enhancing the mineralization rate of organic matter by soil microorganisms (Qiao et al., 2018; Wang and Tang, 2018; Asghar and Kataoka, 2021), improving soil fertility (Li et al., 2016), and ultimately promoting crop growth (Manici et al., 2018). We speculated that it may be because white clover straw return could provide more nutrients to plants for better growth of goosegrass seedlings under white clover treatments at high ratios compared to the ryegrass treatments.

A limitation of this study is the inability to convert the extract concentrations in the germination experiment to the corresponding straw-to-soil ratios in the pot experiment. Another limitation is that we only considered the effects of green manure return on seed germination and seedling growth of goosegrass without considering their effects on the soil. The main mechanism that inhibits the seedling growth of goosegrass remains unclear.

## 5 Conclusion

This study indicated that incorporating white clover and ryegrass as green manure into soil could suppress goosegrass growth. It is necessary to study the direct and indirect effects of decomposition residues on the soil to investigate how green manure residue decomposition inhibits weed growth. For instance, further exploration of the changes in soil composition, nutrients, and microorganisms after the decomposition of straw return in different biomass would help provide a more scientific basis for reducing the usage of herbicides and utilizing white clover or ryegrass returned to the field to manage weeds in agricultural fields.

## Data availability statement

The original contributions presented in the study are included in the article/**Supplementary Material**. Further inquiries can be directed to the corresponding authors.

## Author contributions

YZ: Data curation, Formal analysis, Visualization, Writing – original draft. SL: Conceptualization, Data curation, Methodology, Writing – original draft. XD: Data curation, Writing – original draft. ZC: Data curation, Writing – original draft. ZM: Funding acquisition, Supervision, Validation, Writing – review & editing, Resources. YM: Conceptualization, Funding acquisition, Supervision, Validation, Writing – review & editing, Resources.
