# Supplementary material for: The inhibitory potential of green manure return on the germination and seedling growth of Eleusine indica L
Source: Front Plant Sci. 2024 Feb 7;15:1287379. doi: 10.3389/fpls.2024.1287379 (PMC10879556; doi:10.3389/fpls.2024.1287379)
Supplement: Supplementary file 1 [file Table_1.docx]

Table S1 The list of abbreviations

| Abbreviation type | | Abbreviation meaning |
| --- | --- | --- |
| Treatment | WC | White clover |
|  | RG | Ryegrass |
|  | AE | Aqueous extract |
|  | DL | Decomposed liquid |
|  | SSR | The straw-to-soil ratio |
| Germination experiment test items | Gp | Germination rate |
|  | Gr | Germination potential |
|  | Gi | Germination index |
|  | Vi | Vital index |
|  | PH | The plant height |
|  | FW | The fresh weight |
|  | RI | Response index |
| Pot experiment test items | SPAD | Soil and Plant Analyzer Development |
|  | Pn | The net photosynthetic rate |
|  | Tr | The transpiration rate |
|  | Ci | The stomatal conductance |
|  | Gs | The intercellular carbon dioxide concentration |
|  | Fv/Fm | The maximal quantum yield of photosystem |
|  | ETR | The electron transport rate |
|  | ΦPSII | The quantum yield of photosystem II |
|  | qP | The photochemical quenching |
|  | NPQ | The non-photochemical quenching |
|  | SOD | The superoxide dismutase |
|  | POD | The peroxidase |
|  | CAT | The catalase |
|  | SP | The soluble protein |
|  | MDA | The malondialdehyde |
|  | PCA | Principal component analysis |
